# Supplementary material for: The ITS analysis and identification of Actinidia eriantha and its related species
Source: PLoS One. 2022 Sep 22;17(9):e0274358. doi: 10.1371/journal.pone.0274358 (PMC9498973; doi:10.1371/journal.pone.0274358)
Supplement: Attachment — Submitted filename: Response to Reviewers.docx [file pone.0274358.s002.docx]

Comments and response

1. why did you prefer ITS although discriminations power of SSR are more accurate? is it less expensive to do a sequencing analysis rather genotyping?

**Response**: SSR has some shortcomings: 1) the detection of SSR polymorphism largely depends on the effect of PCR amplification; 2) The existing SSR markers are limited in number, and can not mark all functional genes; 3) The development and synthesis of primers are expensive, difficult and time-consuming.

2. please provide some photos also for the difficult part of morphological description

**Response**: We add Leaf image of 8 species of Actinidia in manuscript for the difficult part of morphological description.

3. finally the manuscript needs to be critically proof read before resubmitted.

**Response**: Dr. Bin Wu of Zhejiang University thoroughly copyedit manuscript for language usage, spelling, and grammar.

4. In your Methods section, please provide additional information regarding the permits you obtained for the work. Please ensure you have included the full name of the authority that approved the field site access and, if no permits were required, a brief statement explaining why.

**Response**: This research material is plant leaves , it is not involve ethical protection and genetic information protection, so no additional information regarding the permits for the work we need.

5. In your Data Availability statement, you have not specified where the minimal data set underlying the results described in your manuscript can be found.

**Response**: The minimal data set underlying the results described in my manuscript can be found in Genbank(accession numbers see S1 Table).
